# Weight gain and comorbidities associated with oral second-generation antipsychotics: analysis of real-world data for patients with schizophrenia or bipolar I disorder

**DOI:** 10.1186/s12888-022-03758-w

**Published:** 2022-02-14

**Authors:** Michael J. Doane, Leona Bessonova, Haley S. Friedler, Kathleen M. Mortimer, Harry Cheng, Thomas Brecht, Amy K. O’Sullivan, Hannah Cummings, David McDonnell, Jonathan M. Meyer

**Affiliations:** 1 Alkermes, Inc., 825 Winter St., Waltham, MA 02451-1420 USA; 2 OMI, Inc., Boston, MA USA; 3 Alkermes Pharma Ireland Ltd., Dublin, Ireland; 4 Department of Psychiatry, University of California San Diego School of Medicine, CA La Jolla, USA

**Keywords:** Cardiometabolic burden, Claims data, Electronic medical record, Treatment patterns

## Abstract

**Background:**

Many second-generation antipsychotics (SGAs) are associated with weight gain and cardiometabolic effects. Antipsychotic-associated weight gain is linked to treatment interruptions, potentially increasing risk of relapse and hospitalization. This retrospective study assessed clinically significant weight gain (CSWG), treatment interruptions, and development of cardiometabolic conditions in patients with schizophrenia (SZ) or bipolar I disorder (BD-I) following initiation of oral SGAs with moderate to high weight gain risk.

**Methods:**

Patients with no prior use of moderate to high weight gain risk oral SGAs were identified from patient-level medical/pharmacy claims and electronic medical records (January 2013–February 2020; OM1 Real-World Data Cloud). Those with ≥ 1 weight measurement in both the 12 months preceding and 3 months after SGA initiation (index date) were analyzed for continuous changes in weight, CSWG (≥ 7% and ≥ 10% increases from baseline), treatment interruptions (switches/discontinuations), and development of cardiometabolic conditions.

**Results:**

Median follow-up times in the SZ (*n* = 8174) and BD-I (*n* = 9142) cohorts were 153.4 and 159.4 weeks, respectively; 45.5% and 50.7% were obese at baseline. Mean (SD) percent weight increase during treatment was 3.3% (7.2) and 3.7% (7.0) for patients with SZ and BD-I, respectively, and was highest for underweight/normal weight patients (SZ: 4.8% [8.1]; BD-I: 5.5% [8.7]). More than 96% had treatment interruptions during follow-up, primarily discontinuations. CSWG and treatment interruptions occurred within a median of 13 and 14 weeks after treatment initiation, respectively. Of patients with CSWG and treatment interruptions, approximately 75% did not return to baseline weight during follow-up. Among those without baseline cardiometabolic conditions, 14.7% and 11.3% of patients with SZ or BD-I, respectively, developed ≥ 1 condition over 12 months post-index. Incidence was generally highest among those who were overweight/obese at baseline and those who experienced CSWG.

**Conclusions:**

In this analysis of real-world data, both weight gain and treatment interruptions occurred early in treatment for patients with SZ or BD-I. Treatment-associated weight gain persisted despite switching or discontinuing index treatment. Additionally, cardiometabolic morbidity increased within 12 months of treatment initiation. Patients with SZ or BD-I are at greater risk than the general population for cardiometabolic conditions; weight gain associated with SGAs may exacerbate these health risks.

## Background

Antipsychotic medications are effective options for the treatment of schizophrenia (SZ) and bipolar I disorder (BD-I) [1, 2], conditions for which lifelong pharmacotherapy is recommended to prevent relapse. Although second-generation antipsychotics (SGAs) have lower extrapyramidal symptom liability than first-generation antipsychotics (FGAs) [3], many SGAs are associated with clinically significant weight gain (CSWG) and increased risks of cardiometabolic effects, including diabetes and cardiovascular disease [2, 4]. Both SZ and BD-I have been associated with the risk of obesity for a variety of reasons, including genetic and lifestyle factors, that act independently of medication effects [5–12]. Additionally, when treated with SGAs, up to 50% of patients may experience CSWG of ≥ 7% of their baseline body weight. Furthermore, CSWG is even more common in patients who are initiating treatment with an SGA for the first time (up to 70%) [13] and in those who are underweight or normal weight at the time of SGA initiation (up to 55%) [14]. Antipsychotic-associated weight gain begins during the first weeks of treatment and may continue over long-term treatment with some SGAs [15]. Additionally, reports of metabolic dysregulation during treatment with some SGAs, including incident hyperglycemia, diabetes mellitus, diabetic ketoacidosis, and dyslipidemia, support a link between treatment and cardiometabolic sequelae [16, 17].

Weight gain and metabolic effects of SGA treatment can be distressing to patients [18, 19]. For example, two recent surveys found that weight gain was among the most common and the most bothersome side effects that patients with SZ [20] or BD-I [21] experienced during antipsychotic treatment. Weight gain and metabolic effects were also among the leading reasons for suboptimal adherence with, and treatment discontinuation of, oral SGAs in the Clinical Antipsychotic Trials of Intervention Effectiveness (CATIE) study [3]. Both of these outcomes increase the risk of relapse and hospitalization [18, 22] with associated increases in direct medical costs [23].

Existing data on antipsychotic-associated weight gain are mostly derived from clinical trials, and there is a lack of longitudinal data on weight gain among patients treated with SGAs in real-world settings. This retrospective analysis used a combination of medical and pharmaceutical claims and electronic medical record (EMR) data to describe real-world patterns of antipsychotic-associated weight gain and other outcomes among patients with SZ or BD-I following initiation of select oral SGAs associated with moderate to high risk of weight gain. Specifically, these analyses describe the proportion of patients who develop CSWG or interrupt treatment (switches or discontinuations) as well as the time course over which these events occur. Additionally, we assessed CSWG in relation to (1) duration of antipsychotic treatment; (2) baseline body mass index (BMI); (3) weight trajectory following treatment interruption; and (4) the development of new cardiometabolic conditions in patients with and without preexisting conditions at baseline.

## Methods

### Data source

This retrospective, observational, longitudinal analysis used real-world data from the OM1 Real-World Data Cloud, which integrates de-identified patient-level health care claims and EMR data from more than 50 million patients, using patient-specific identifiers. Administrative claims data included data from commercial insurers, Medicare, and Medicaid; EMR data were derived from several health care delivery and EMR provider systems. Medical and/or pharmacy claims contained billing and coding history on inpatient/outpatient encounters from acute care facilities, ambulatory surgery centers, and clinics. EMR data sources provided laboratory data, vital signs, problem lists, and other clinical details unavailable in claims data alone. Claims and EMR data were updated at least quarterly. All database records were de-identified and certified to be fully compliant with US patient confidentiality requirements set forth in the Health Insurance Portability and Accountability Act (HIPAA) of 1996. Because this study used only de-identified patient records and did not involve the collection, use, or transmittal of individually identifiable data, it was exempt from institutional review board approval. This analysis covered the time from January 2013 to February 2020.

### Patients

The study included patients from the OM1 Real-World Data Cloud database who initiated select oral SGAs associated with moderate to high risk of weight gain and whose records indicated a diagnosis of SZ and/or BD-I, with no prior observed use of these SGAs or FGAs. Patients were included in this analysis if they were ≥ 18 years of age at index and had a diagnosis of SZ or BD-I prior to or within 30 days after initiating treatment. The diagnosis of SZ or BD-I was determined by having at least 1 diagnostic code occurring within 30 days prior to or on the index date, per the International Classification of Diseases 9th or 10th revision, Clinical Modification (ICD-9-CM/ICD-10-CM) or Systemized Nomenclature of Medicine – Clinical Terms (SNOMED CT) diagnostic codes for the respective condition. Patients with a history of both SZ and BD-I diagnoses were categorized in the SZ cohort. The index date was defined as the date of a new prescription/fill of one of the SGAs associated with moderate to high risk of weight gain indicated for these conditions, with no observed prior use of these respective SGAs, or of any FGAs. Patients were required to have linked EMR and claims data for ≥ 12 months before and ≥ 12 months after the index date. Patients also had to have ≥ 1 weight measurement in the 12 months prior to or on the index date and ≥ 1 weight measurement in the 3 months following the index date.

**Fig. 1:**
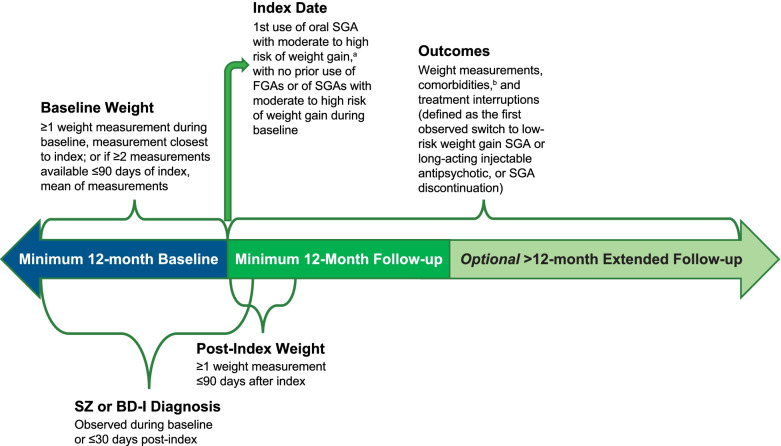
Study Schema. ^a^Included clozapine, iloperidone, and paliperidone (SZ only); olanzapine, risperidone, quetiapine (SZ or BD-I); and olanzapine/fluoxetine (BD-I only). ^b^Comorbidities were only assessed during the baseline and 12-month post-index periods. BD-I, bipolar I disorder; FGA, first-generation antipsychotic; SGA, second-generation antipsychotic; SZ, schizophrenia

Patients who initiated moderate to high weight gain risk SGAs were followed for a minimum of 12 months (Fig. 1). Based on a comprehensive review of antipsychotic-related side effects from clinical studies [4], SGAs, defined as having a moderate to high risk of weight gain, included clozapine, iloperidone, and paliperidone (each approved for SZ), olanzapine/fluoxetine (approved for depressive episodes in BD-I [24]), and olanzapine, risperidone, and quetiapine (each approved for SZ and BD-I). SGAs associated with a low risk of weight gain (ie, amisulpride, aripiprazole, brexpiprazole, cariprazine, lurasidone, and ziprasidone) [4] were not included as SGA initiation treatments in this study, but were included in descriptive analyses as a category of antipsychotics to which patients may have switched.

### Outcomes

Baseline weight was defined as the measurement closest to or on the index date. If multiple weight measurements were available within 90 days prior to and including the index date, these weights were averaged to calculate baseline weight. During the follow-up period, percent change from baseline weight and the proportions of patients with CSWG of ≥ 7% and ≥ 10% from baseline were determined.

Treatment interruptions were defined as medication switches or discontinuations. A switch was defined as any new prescription/fill for an oral SGA not associated with a moderate to high risk of weight gain (ie, SGAs with a low risk of weight gain). Additionally, initiation of a long-acting injectable antipsychotic during the index medication era, or within 30 days of the end of the index medication era, was considered a medication switch. The switch date was based on the prescription/fill date and number of days’ supply for the new antipsychotic. Use of another SGA with moderate to high weight gain risk indicated for the same condition was not categorized as a switch, and patients were considered to be on continuous treatment within this group of medications. Discontinuations were defined to have occurred if no additional prescriptions/fills for a moderate to high weight gain risk SGA were recorded within 30 days of the estimated last day of index medication use.

The prevalence of cardiometabolic conditions at baseline was determined for all patients. Patients were classified as having a cardiometabolic condition of interest if they had a combination of at least 2 relevant ICD-9-CM/ICD-10-CM or SNOMED CT diagnostic codes, at least 30 days apart. Key cardiometabolic conditions of interest included coronary artery disease, type 2 diabetes mellitus, dyslipidemia, and hypertension. Other cardiometabolic conditions included those not defined as key cardiometabolic conditions (eg, acute events, congestive heart failure, or other). The incidence of cardiometabolic conditions during the 12-month post-index period was assessed in patients without these conditions at baseline. In addition, increased cardiometabolic burden (ie, development of a new cardiometabolic condition in patients who had prevalent cardiometabolic conditions at baseline) was assessed over the 12-month post-index period.

### Analytic methods

Analyses were summarized separately for SZ and BD-I cohorts. Descriptive analyses included proportions of patients with CSWG and treatment interruptions, as well as median time to each outcome. Categorical variables were presented as numbers and percentages; continuous variables were presented as means, standard deviations (SDs), medians, and 25th (Q1) and 75th (Q3) percentiles. Kaplan-Meier models were used to estimate time to CSWG (≥ 7% and ≥ 10%). Additional analyses examined percent change in weight, stratified by baseline BMI; assessments of cardiometabolic conditions were stratified by baseline BMI and CSWG.

## Results

### Patient characteristics

A total of 8174 patients with SZ and 9142 patients with BD-I initiated oral SGAs with moderate to high weight gain risk and were included in this study. The median length of follow-up was 153.4 and 159.4 weeks for the SZ and BD-I cohorts, respectively. The mean patient age at initiation was 57.4 years in the SZ cohort and 48.2 years in the BD-I cohort, with 36.1% and 15.5% of patients aged ≥ 65 years, respectively; most patients were female (SZ, 61.6%; BD-I, 72.2%) and white (SZ, 80.9%; BD-I, 89.7%; Table 1). Based on BMI, 45.5% and 50.7% of patients with SZ and BD-I, respectively, were classified as obese (BMI ≥30.0 kg/m^2^) at baseline. Other than SGAs, the 3 most common psychotropic medications used at baseline in the SZ and BD-I cohorts, respectively, were antidepressants (62.8% and 69.7%), anti-anxiety medications (38.2% and 46.4%), and mood stabilizers (16.8% and 23.1%). Among patients with known insurance (SZ, 84.2%; BD-I, 78.7%), commercial insurance was the most common source of coverage (SZ, 40.4%; BD-I, 51.1%).

**Table 1:** Patient Characteristics at Index Date

Parameter	Patients With SZ (***n*** = 8174)	Patients With BD-I (***n*** = 9142)
Sex, n (%)		
Female	5037 (61.6)	6604 (72.2)
Race, n (%)^a^		
White	5554 (80.9)	7036 (89.7)
Black	1235 (18.0)	770 (9.8)
Other	75 (1.1)	37 (0.5)
Age at index, mean (SD), years	57.4 (17.7)	48.2 (15.3)
Age category at index, n (%), years		
18–24	354 (4.3)	634 (6.9)
25–34	656 (8.0)	1359 (14.9)
35–44	908 (11.1)	1680 (18.4)
45–54	1515 (18.5)	2111 (23.1)
55–64	1788 (21.9)	1942 (21.2)
≥ 65	2953 (36.1)	1416 (15.5)
Insurance type, n (%)^b^		
Commercial	2781 (40.4)	3677 (51.1)
Medicaid	773 (11.2)	939 (13.1)
Medicare	2392 (34.7)	1742 (24.2)
Multiple	909 (13.2)	775 (10.8)
Other	31 (0.5)	59 (0.8)
Comorbidities, n (%)^c^		
Depression	4208 (51.5)	4883 (53.4)
Anxiety disorders	3988 (48.8)	5142 (56.2)
Chronic pulmonary disease	3597 (44.0)	3943 (43.1)
Diabetes without chronic complications	2253 (27.6)	1787 (19.5)
Cerebrovascular disease	1884 (23.0)	1093 (12.0)
Congestive heart failure	1132 (13.8)	615 (6.7)
Peripheral vascular disease	1048 (12.8)	623 (6.8)
Myocardial infarction	592 (7.2)	389 (4.3)
Current tobacco use	3667 (44.9)	4442 (48.6)
Weight, mean (SD), kg	85.05 (23.5)	87.55 (24.1)
BMI category, n (%)^d^		
< 18.5 kg/m^2^	168 (2.1)	142 (1.6)
18.5–24.9 kg/m^2^	1968 (24.3)	1924 (21.1)
25.1–29.9 kg/m^2^	2277 (28.1)	2425 (26.6)
≥ 30.0 kg/m^2^	3690 (45.5)	4619 (50.7)
SGA treatment, n (%)		
Olanzapine	1513 (18.5)	1564 (17.1)
Clozapine	236 (2.9)	N/A
Iloperidone	38 (0.5)	N/A
Paliperidone	193 (2.4)	N/A
Risperidone	2246 (27.5)	1633 (17.9)
Quetiapine	4038 (49.4)	5935 (64.9)
Olanzapine/fluoxetine combination	N/A	69 (0.8)

^a^Total *n* = 6864 for SZ, *n* = 7843 for BD-I

^b^Total *n* = 6886 for SZ, *n* = 7192 for BD-I

^c^The 3 comorbidities with the highest prevalence for each patient cohort (depression, anxiety disorders, and chronic pulmonary disease) are shown, in addition to select other cardiovascular and diabetes comorbidities

^d^Total, *n* = 8103 for SZ, *n* = 9110 for BD-I

BD-I, bipolar I disorder; BMI, body mass index; N/A, not applicable; SD, standard deviation; SZ, schizophrenia

### Weight gain after initiation of oral SGAs associated with moderate to high risk of weight gain

During the post-index follow-up period, a mean (SD) of 14.7 (13.5) and 15.4 (13.5) weight measures were taken in the SZ and BD-I cohorts, respectively. For both cohorts, the mean duration of treatment was approximately 30 weeks. The mean (SD) percent increase in weight during treatment with index SGAs of moderate to high weight gain risk was 3.3% (7.2) and 3.7% (7.0) for the SZ and BD-I cohorts, respectively. A total of 21.1% and 22.3% of patients with SZ and BD-I, respectively, had ≥ 7% weight gain from baseline during treatment.

To understand how baseline BMI may impact weight gain during follow-up, weight gain during both the index treatment period and the total follow-up period were assessed stratified by baseline BMI category. Underweight/normal weight patients experienced the highest percent weight gain during treatment (SZ, 4.8% [8.1]; BD-I, 5.5% [8.7]) compared with patients who were overweight (SZ, 3.4% [7.6]; BD-I, 3.8% [6.8]) or obese (2.3% [6.0]; BD-I: 2.7% [5.9]). A higher proportion of patients who were underweight/normal weight at baseline had CSWG of ≥ 7% during treatment (SZ, 29.1%; BD-I, 31.7%) compared with patients who were overweight at baseline (SZ, 22.2%; BD-I, 23.9%) or obese (SZ, 15.5%; BD-I, 17.2%).

**Fig. 2:**
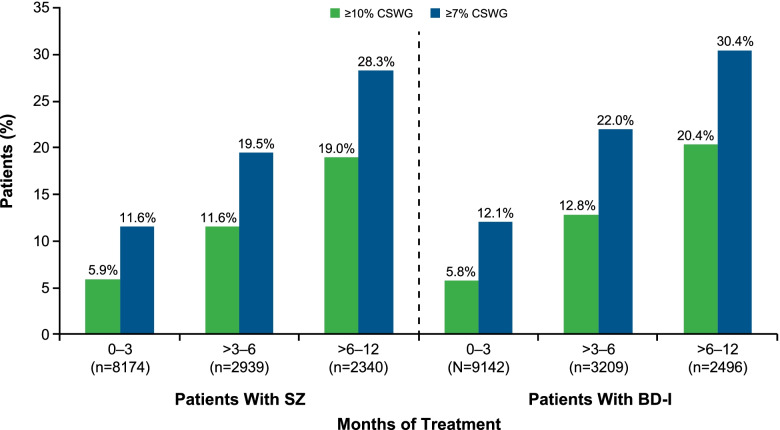
Proportion of Patients With CSWG During Treatment With Oral SGAs of Moderate to High Weight Gain Risk. BD-I, bipolar I disorder; CSWG, clinically significant weight gain; SGA, second-generation antipsychotic; SZ, schizophrenia; Tx, treatment

Weight gain was most pronounced during treatment: while time on index treatment represented approximately 20% of patients’ total follow-up time, approximately 40% of the observed weight gain during all of follow-up occurred during patients’ time on treatment. A subgroup analysis examined the proportion of patients who developed CSWG in the 12-month post-index period by time on treatment. The proportion of patients who developed CSWG from baseline increased with the duration of treatment with moderate to high weight gain risk SGAs in both the SZ and the BD-I cohorts, as shown in Fig. 2. The median time to CSWG of ≥ 7% was 14 weeks (for both cohorts); median time to CSWG of ≥ 10% was 19 weeks (SZ cohort) and 20 weeks (BD-I cohort). In both cohorts, the median time to ≥ 7% or ≥ 10% CSWG was similar across baseline BMI categories.

### Weight gain and treatment interruptions during use of oral SGAs associated with a moderate to high risk of weight gain

More than 96% of patients had a treatment interruption during follow-up (SZ, 7920/8174 [96.9%]; BD-I, 8915/9142 [97.5%]). The majority of treatment interruptions were discontinuations without switches to SGAs with low weight gain risk or long-acting injectable antipsychotics (SZ, 94.9%; BD-I, 94.4%). The median (Q1–Q3) duration of treatment was 13 (4 − 41) weeks and 13 (4 − 39) weeks, respectively, for the SZ and BD-I cohorts. Among patients in the SZ cohort who had a treatment interruption, 20.2% and 12.7% experienced CSWG of ≥ 7% and ≥ 10%, respectively, before treatment interruption; the median (Q1 − Q3) times to CSWG of ≥ 7% and ≥ 10% were 12 (5–28) weeks and 18 (7–37) weeks, respectively. In the BD-I cohort, 21.5 and 12.9% of patients experienced CSWG of ≥ 7% and ≥ 10%, respectively, before treatment interruption; the median (Q1 − Q3) times to CSWG of ≥ 7% and ≥ 10% were 13 (5 − 30) weeks and 19 (8 − 40) weeks, respectively.

**Fig. 3:**
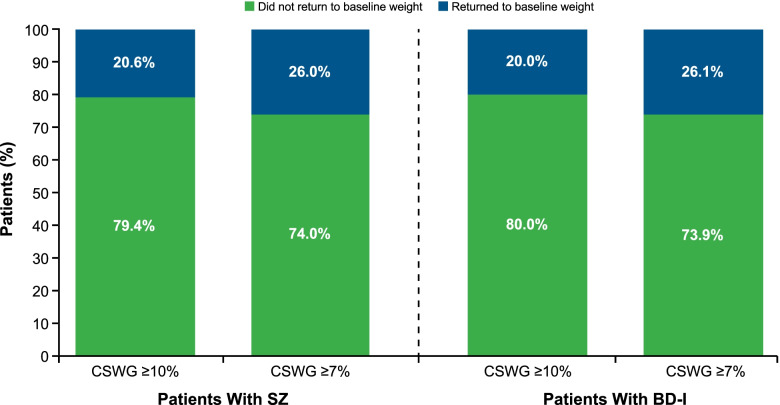
Return to Baseline Weight After Treatment Interruption With Oral SGAs Associated With Moderate to High Risk of Weight Gain. BD-I, bipolar I disorder; CSWG, clinically significant weight gain; SGA, second-generation antipsychotic; SZ, schizophrenia

The majority of patients with CSWG of ≥ 7% (SZ, 74.0%; BD-I, 73.9%) or ≥ 10% (SZ, 79.4%; BD-I 80.0%) who experienced treatment interruptions, including those who switched to an SGA with a low risk of weight gain or long-acting injectable antipsychotic, did not return to their baseline weight during the follow-up period (Fig. 3). Among those patients with CSWG at any level who then returned to their baseline weight or lower after treatment interruption, the median time to weight reduction was 38 to 39 weeks in the SZ cohort and 39 to 47 weeks in the BD-I cohort.

### Baseline prevalence and development of new cardiometabolic conditions within 12 months of index antipsychotic treatment initiation

**Fig. 4:**
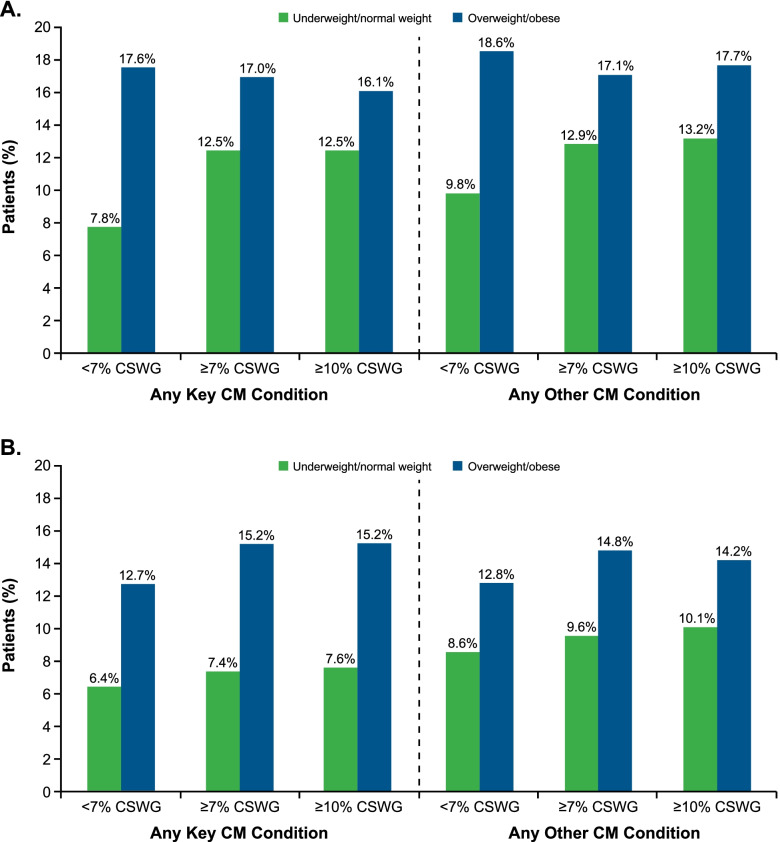
Incidence of Key/Other Cardiometabolic Conditions by BMI in the 12 Months Post-Index in Patients With No Cardiometabolic Conditions at Baseline. (A) Patients With SZ, and (B) Patients With BD-I. BD-I, bipolar I disorder; BMI, body mass index; CM, cardiometabolic; CSWG, clinically significant weight gain; SZ, schizophrenia

At baseline, 1984 (24.3%) and 3542 (38.7%) patients in the SZ and BD-I cohorts, respectively, had no diagnostic codes indicative of key cardiometabolic conditions of interest (coronary artery disease, type 2 diabetes mellitus, dyslipidemia, hypertension), and 1964 (24.0%) and 3613 (39.5%) patients, respectively, had no diagnostic codes indicative of the presence of any other cardiometabolic conditions (eg, acute events, congestive heart failure). Among patients with no cardiometabolic conditions at baseline, key cardiometabolic conditions developed in 14.7% in the SZ cohort and in 11.3% in the BD-I cohort. Cardiometabolic conditions other than the key conditions of interest developed in 15.8% and 12.0% of the SZ and BD-I cohorts, respectively. In both cohorts, the incidence of key or other cardiometabolic conditions was higher among patients who were overweight/obese at baseline than underweight/normal weight patients (SZ: key/other cardiometabolic events, 17.4%/18.2% vs 10.1%/11.3%; BD-I: key/other cardiometabolic events, 13.5%/13.3% vs 6.9%/9.0%). Additionally, in those patients without cardiometabolic conditions at baseline, the incidence of key or other cardiometabolic conditions during the 12-month post-index period was numerically higher in patients with CSWG versus those without CSWG (both cohorts), although the association was less pronounced in underweight/normal weight patients with BD-I (Fig. 4).

**Fig. 5:**
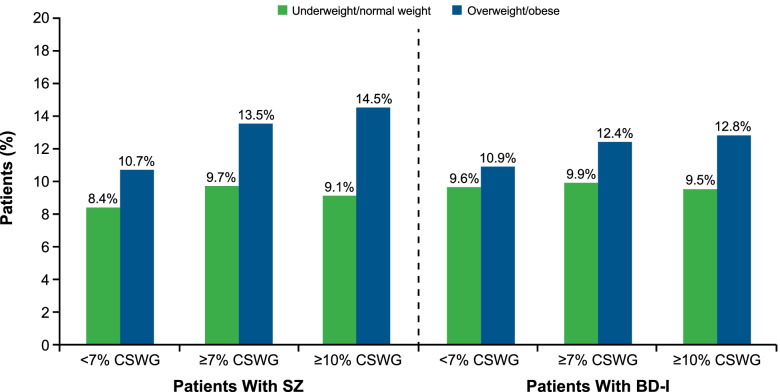
Increased Cardiometabolic Burden by CSWG in the 12 Months Post-Index in Patients With Cardiometabolic Conditions at Baseline. BD-I, bipolar I disorder; CSWG, clinically significant weight gain; SZ, schizophrenia

In total, 6742 (82.5%) of the SZ cohort and 6323 (69.2%) of the BD-I cohort had a prevalent cardiometabolic condition (key or other) at baseline. Of those with prevalent baseline cardiometabolic conditions, 10.7% of patients with SZ and 11.0% of those with BD-I developed a new cardiometabolic condition during the 12-month post-index period. The incidence of new key cardiometabolic conditions was higher among patients who were overweight/obese than in those who were underweight/normal weight at baseline in the SZ cohort (11.3% vs 8.9%) and in the BD-I cohort (11.3% vs 9.7%), respectively. Additionally, the incidence of new key cardiometabolic conditions was higher among patients with CSWG compared with those without CSWG (both cohorts); the trend was strongest among overweight/obese patients at baseline versus those who were underweight/normal weight at baseline (Fig. 5).

## Discussion

Results of this real-world, longitudinal, retrospective analysis of weight gain during antipsychotic treatment suggest that the use of SGAs with a moderate to high risk of weight gain is associated with CSWG for patients with either SZ or BD-I. Weight gain occurred early after initiating SGAs and persisted among patients with SZ or BD-I, even after discontinuing treatment. Notably, approximately 75% of patients with ≥ 7% weight gain and approximately 80% with ≥ 10% CSWG did not return to their baseline weight despite switching or discontinuing treatment; for those who did return to their baseline weight, it took a median of 38 weeks for those with ≥ 7% CSWG and 47 weeks for those with ≥10% CSWG to do so. Furthermore, during follow-up, more than 96% of patients in each diagnostic group had a treatment interruption, most of which were discontinuations of medication without timely switches to new medications.

The weight profile of patients in this study is similar to those described in previous research. The prevalence of obesity has been estimated to be as high as 55% and 49% in SZ and BD-I populations, respectively [25]; in this study, 45.5% of patients with SZ and 50.7% of patients with BD-I were classified as obese before treatment initiation, consistent with the published literature. Patients have an increased risk of obesity attributable not only to the use of antipsychotic medication, but also genetic predisposition, dietary factors, sedentary lifestyle, and/or tobacco or substance use [11, 12]. Other patient characteristics that have been associated with susceptibility to antipsychotic weight gain include younger age, female sex, and non-white race [26, 27]. Low baseline BMI is a known risk factor for antipsychotic-induced weight gain [25], although an association between greater weight gain and increased BMI at baseline [26] and a lack of any association between baseline BMI and weight gain with antipsychotic treatment [28] have also been reported. In these analyses, initiation of moderate to high weight gain risk SGAs was associated with weight gain, regardless of baseline BMI, but the percent increase in weight was highest among those who were underweight or normal weight at baseline, consistent with some earlier findings. Together, these results suggest that patients with a lower BMI, including younger patients or patients in the early phase of illness, should be monitored closely for weight gain, and that initial treatment choices with a lower risk of weight gain may be preferred in this patient population.

Most patients who experienced CSWG and had an interruption in treatment did not return to their baseline weight. Of the minority of patients with CSWG during treatment who did return to their baseline weight, reversal of weight gain took a median time of 9 months or longer. In a population of patients who require long-term treatment of a chronic mental health disorder, the observed persistence of weight gained during treatment may represent a clinical liability contributing to suboptimal adherence and poor patient outcomes. Notably, weight gain is associated with reduced adherence [18, 29], which in turn increases the risk of relapse, hospitalization, and disease progression [22].

A major health risk related to weight gain is the development of cardiovascular disease [12, 30, 31]. Treatment initiation with moderate to high weight gain risk SGAs was associated with incident cardiometabolic conditions within the first 12 months of treatment in both SZ and BD-I cohorts. Most patients in this analysis had ≥ 1 cardiometabolic comorbidity at baseline. After initiation of SGAs with moderate to high risk of weight gain, approximately 11% of these patients with preexisting conditions developed a new cardiometabolic condition, suggesting a further decline in cardiometabolic health and an increased burden of illness(es). These findings were especially pronounced for the SZ cohort, those who were overweight or obese at baseline, and those who developed CSWG in the post-index period, suggesting that overweight/obese patients with preexisting cardiometabolic conditions are another patient population in whom the use of SGAs with moderate to high risk of weight gain should be carefully evaluated.

Among the minority of patients in the SZ and BD-I cohorts without a cardiometabolic condition at the time of treatment initiation, rates of cardiometabolic conditions within the 12 months after initiation of SGAs with moderate to high risk of weight gain were numerically highest in those who were overweight or obese at baseline. Rates of cardiometabolic conditions within the 12 months after initiation were also numerically highest for patients with CSWG, except among patients in the SZ cohort who were overweight or obese at baseline. These results indicate that patients without cardiometabolic comorbidities are at risk for developing one or more conditions after initiating treatment with moderate to high risk of weight gain SGAs, especially among patients who are already overweight or obese.

### Limitations

There are limitations inherent to retrospective study designs and the use of secondary data. Patients providing data for this analysis were predominantly white and female, despite past reports that the 12-month prevalence of SZ and BD is similar between male and female patients [32, 33], that the prevalence of self-reported psychosis is higher in black patients compared with white patients [34], and that there are no significant differences in prevalence of BD across race [35]. It should be noted that the EMR and claims data captured for this analysis were contingent on the characteristics of patients who interacted with and utilized health care services. These disparities may be expected given that black and/or male patients tend to use fewer mental health services compared with their white and female counterparts, despite similar rates of serious mental illness [36, 37]. Therefore, findings from the current study in which female and white patients are more prevalent, may not be generalizable to the full patient population with SZ or BD-I. Additionally, the use of real-world data may not precisely capture the timing of weight gain and therefore may underestimate possible associations between weight gain and other study outcomes. Some weight measurements may have been recorded based on patient self-report rather than being measured by a physician. While previous research has shown that olanzapine and clozapine are associated with the greatest amount of antipsychotic-associated weight gain [4], the results herein do not appear to be driven by treatment with these two agents alone, given their overall use in patients (Table 1) contributing data for this analysis. Because oral SGAs and long-acting injectable antipsychotics have different adherence and discontinuation patterns [38], initiating a long-acting injectable antipsychotic was considered a discontinuation, even if switching to the same antipsychotic. Although few patients (≤1.3%) switched to long-acting injectable antipsychotics, this may have contributed to an overall underestimation of the magnitude of CSWG. Furthermore, the exact timing of medication discontinuations could not be determined because the duration of medication use was defined by days of supply, potentially overestimating the overall time to discontinuation. The analysis of comorbidities was limited to the 12-month post-index period only, and the long-term impact of CSWG on cardiometabolic conditions could not be assessed. In addition, because data were obtained from multiple sources, not all patients may have had complete data from all sources during the time frame of this study. Although medical claims data were required for all patients, it is possible that not all comorbid conditions were captured within this data source; thus, the incidence and increased burden of cardiometabolic comorbidities may have been underestimated.

This study also had a higher proportion of commercially insured patients compared with other published data on adults with SZ [39], and may reflect a more stable or well-managed population that may not be representative of all patients. Lastly, the mean patient age in this sample was older than 50 years in both cohorts, which is 11 to 14 years older than that reported in previous studies [40, 41], so certain findings may not be generalizable to younger populations.

## Conclusions

The use of SGAs with moderate to high risk of weight gain is associated with weight gain early in treatment, with a similar incidence of CSWG in patients with SZ or BD-I. Most patients with CSWG do not return to baseline weight after switching or discontinuing treatment. Consistent with previous research, weight gain during treatment with SGAs of moderate to high weight gain risk was highest in those who were underweight or of normal weight at baseline, although patients who were overweight or obese upon initiating index treatments also experienced weight gain on average. Treatment discontinuations were common, with more than 96% of patients discontinuing treatment during follow-up. Roughly 11% to 15% of patients with no key cardiometabolic conditions at baseline went on to develop these conditions in the post-index period. In patients with a baseline cardiometabolic condition, increased cardiometabolic burden was noted in both patient cohorts during treatment with SGAs known to be associated with a moderate to high risk of weight gain, especially in patients who were obese or overweight at baseline.

Overall, findings suggest that the initiation of SGAs that are effective in the symptomatic control of SZ and BD-I needs to be balanced with the side effect burden profile of the agent in question, as there may be both short- and long-term consequences of significant weight gain, reduced persistence on treatment, and the onset of new cardiometabolic conditions, resulting in poorer clinical outcomes, including relapse, rehospitalization, and development or worsening of cardiometabolic comorbidities.

## Data Availability

Alkermes, Inc., is committed to public sharing of data, in accordance with applicable regulations and laws. Data may be subject to third-party rights and restrictions. Please contact the corresponding author with any questions.

## Abbreviations

BD-I: Bipolar I disorder
BMI: Body mass index
CSWG: Clinically significant weight gain
EMR: Electronic medical record
FGA: First-generation antipsychotic
ICD-9-CM/ICD-10-CM: International Classification of Diseases, 9th or 10th revision, Clinical Modification
Q: Quartile
SD: Standard deviation
SGA: Second-generation antipsychotic
SNOMED CT: Systemized Nomenclature of Medicine – Clinical Terms
SZ: schizophrenia
